# Acute Myocardial Infarction in Medicare Beneficiaries During and After the COVID-19 Pandemic

**DOI:** 10.1001/jamanetworkopen.2026.4122

**Published:** 2026-04-01

**Authors:** Jonah M. Graves, R. J. Waken, Fengxian Wang, Rishi K. Wadhera, Jose F. Figueroa, E. John Orav, Osvaldo J. Laurido-Soto, Karen E. Joynt Maddox

**Affiliations:** 1 Pulmonary and Critical Care Medicine Division, Department of Medicine, Washington University School of Medicine, St Louis, Missouri; 2 Center for Advancing Health Services, Policy & Economics Research, Washington University School of Medicine, St Louis, Missouri; 3 Division of Biostatistics, Institute for Informatics, Data Science, and Biostatistics, Washington University School of Medicine, St Louis, Missouri; 4 Cardiovascular Division, Department of Medicine, Washington University School of Medicine, St Louis, Missouri; 5 Richard A. and Susan F. Smith Center for Outcomes Research, Boston, Massachusetts; 6 Division of Cardiovascular Medicine, Beth Israel Deaconess Medical Center, Boston, Massachusetts; 7 Harvard Medical School, Boston, Massachusetts; 8 Department of Health Policy & Management, Harvard T. H. Chan School of Public Health, Boston, Massachusetts; 9 Department of Medicine, Brigham and Women’s Hospital, Harvard Medical School, Boston, Massachusetts; 10 Division of General Internal Medicine and Primary Care, Department of Medicine, Brigham and Women’s Hospital, Boston, Massachusetts; 11 Department of Biostatistics, Harvard T. H. Chan School of Public Health, Boston, Massachusetts; 12 Department of Neurology, Washington University School of Medicine, St Louis, Missouri

## Abstract

**Importance:**

During early months of the COVID-19 pandemic, presentations for acute myocardial infarction (AMI) declined significantly, and outcomes worsened. However, the full extent and long-term sequelae of changes in AMI epidemiology during the pandemic remain uncertain, as does whether these patterns differed by rurality.

**Objective:**

To describe the epidemiology of AMI-related hospitalizations, interventions, and outcomes among Medicare beneficiaries throughout the COVID-19 pandemic, focusing on differences in urban and rural populations.

**Design, Setting, and Participants:**

This retrospective cohort study included all Medicare fee-for-service beneficiaries with AMI between January 1, 2018, and December 31, 2023, in the analysis. Data were analyzed from March 19 to July 9, 2025.

**Exposures:**

Time period (prepandemic [January 1, 2018, to December 31, 2019], pandemic [January 1, 2020, to December 31, 2021], and postpandemic [January 1, 2022, to December 31, 2023]) and beneficiary-level rurality.

**Main Outcomes and Measures:**

The primary outcome was in-hospital death, defined as death within 1 day of discharge from the index episode of AMI. Secondary outcomes included death within 90 days of the index admission date and postdischarge outcomes. AMI episodes were defined as any emergency department (ED), observational, or inpatient stay with a primary ST-segment elevation myocardial infarction (STEMI) or non-STEMI (NSTEMI) diagnosis or a primary cardiogenic shock and secondary STEMI or NSTEMI diagnosis. Generalized estimating equations clustering on hospitals were used to compare pandemic and postpandemic outcomes with the prepandemic period, adjusting for beneficiary characteristics.

**Results:**

A total of 1 152 851 AMI episodes among 1 032 212 beneficiaries were identified between 2018 and 2023, of which 75.6% were NSTEMI. Most AMI episodes were among male (57.6%) beneficiaries aged 65 to 80 years (56.8%). The unadjusted quarterly incidence of AMI decreased from 17.2 to 13.0 episodes per million beneficiary days at risk (quarter 1 of 2018 to quarter 4 of 2023). In-hospital (adjusted odds ratio [AOR], 1.09; 95% CI, 1.07-1.11]) and 90-day mortality (AOR, 1.10; 95% CI, 1.09-1.12) increased during the pandemic and then returned to baseline or lower (AORs, 0.99 [95% CI, 0.97-1.01] and 0.96 [95% CI, 0.95-0.98], respectively). After the pandemic, beneficiaries were less likely to discharge to a skilled nursing facility (AOR, 0.67; 95% CI, 0.66-0.68), utilize the ED (adjusted incidence rate ratio [AIRR], 0.93; 95% CI, 0.92-0.94), or experience readmission (AIRR, 0.90; 95% CI, 0.90-0.92) within 90 days of their index episode of AMI. Patterns were largely similar by rurality.

**Conclusions and Relevance:**

In this retrospective cohort study of fee-for-service Medicare beneficiaries, the incidence of AMI decreased during and after the pandemic. Beneficiaries experienced greater in-hospital and 90-day mortality during the pandemic. After the pandemic, in-hospital and 90-day mortality returned to baseline among micropolitan and rural beneficiaries and was lower than baseline among urban beneficiaries.

## Key Points

**Question:**

Were there changes in the incidence or outcomes of acute myocardial infarction (AMI) among Medicare beneficiaries during and after the COVID-19 pandemic, and did these changes differ by rurality?

**Findings:**

In this cohort study of 1 152 851 AMI episodes among all fee-for-service Medicare beneficiaries, the incidence of AMI declined during and after the COVID-19 pandemic. In-hospital mortality increased during the pandemic and decreased back to baseline after the pandemic; urban beneficiaries experienced a greater reduction in mortality after the pandemic.

**Meaning:**

These findings suggest that further work is needed to better understand the changing cardiovascular risk profiles among Medicare beneficiaries and to seek innovative solutions to the disparate outcomes.

## Teaser

This cohort study describes the epidemiology of acute myocardial infarction–related hospitalizations among fee-for-service Medicare beneficiaries and characterizes outcome trends across the COVID-19 pandemic, focusing on differences between urban and rural populations.

## Introduction

During the COVID-19 pandemic, health systems across the US experienced profound disruptions in their usual care delivery, and emergency department (ED) visits and hospitalizations for non–COVID-19 related illnesses, including cardiovascular disease, declined significantly.^1,2,3^ Presentations for acute myocardial infarction (AMI) dropped by as much as half during the early weeks of the pandemic,^4,5,6^ with a lower likelihood of undergoing revascularization among those who did present with AMI.^5,6,7^

Whether trends in AMI-related health care utilization and outcomes have returned to prepandemic levels remains unknown. Further, whether pandemic-related differences varied along geographic lines has not been well delineated, although there is reason to believe that rural individuals might have been harmed to a greater degree both in the short and long terms. Rural patients with AMI were less likely to undergo interhospital transfer during the pandemic,^8^ and higher rates of delayed presentation for ST-segment elevation myocardial infarction (STEMI) were observed among rural patients during the pandemic.^9^ Because mortality from coronary artery disease was higher in rural areas relative to urban areas at baseline, as were rates of smoking, obesity, hypertension, diabetes, and all-cause as well as hypertension- and diabetes-related mortality,^10,11,12,13,14,15^ rural populations might have been more susceptible to the long-term effects of disruptions in hospital services and capacity that took place in 2020.

The goal of this study was to assess the epidemiology of AMI-related hospitalizations among fee-for-service (FFS) Medicare beneficiaries and characterize outcome trends across the pandemic, focusing on differences between urban and rural populations. We hypothesized that rural beneficiaries experienced greater mortality from AMI during the pandemic and that rates did not return to prepandemic levels.

## Methods

This study was deemed exempt from review by the Washington University in St Louis Human Research and Protection Office, which also waived the need for informed consent owing to the deidentified nature of the data. The study followed the Strengthening the Reporting of Observation Studies in Epidemiology (STROBE) reporting guideline.

### Data Source

We performed a retrospective cohort study of all Medicare FFS beneficiaries from January 1, 2018, to December 31, 2023, using beneficiary-level inpatient, outpatient, and postacute claims from the research identifiable files, as well as beneficiary-level demographic information in the Master Beneficiary Summary File and chronic conditions ascertained from prior claims. Data were accessed through the Research Data Assistance Center’s Virtual Research Data Center (VRDC). Data were analyzed from March 19 to July 9, 2025. All analyses and reported results are compliant with the data use agreement outlined by the Research Data Assistance Center VRDC.

First, we defined inpatient claims as any claim from the inpatient claims file, emergency department (ED) claims as any inpatient or outpatient claim with a revenue center code of 045[0-9] or 0981, and observational stay claims as any inpatient or outpatient claim with a revenue center code of 0761. We combined hospitalization claims into a single episode using an algorithm detailed in the eFigure in Supplement 1 characterizing temporally adjacent claims as single, cohesive episodes. We defined an index AMI episode based on the presence of at least 1 ED, observational, or inpatient hospitalization claim with either a primary STEMI or non-STEMI (NSTEMI) diagnosis or a primary diagnosis of cardiogenic shock with a secondary diagnosis of STEMI or NSTEMI, identified using codes from the *International and Statistical Classification of Diseases, Tenth Revision,* (eTable 1 in Supplement 1). Beneficiary files were surveyed for index AMI episodes and subsequent acute care encounters between January 1, 2018, and December 31, 2023. Due to unknown sex codes and invalid or missing Federal Information Processing Standards codes, available on the VRDC, 55.2 and 4 287 135.0 beneficiary-years, respectively, were excluded from surveillance for AMI episodes, representing 1.9% of the 223 078 150.6 beneficiary-years surveyed.

### Outcomes

The primary outcome was in-hospital death, defined as death within 1 day of discharge from the index episode. Secondary outcomes included death within 90 days of the index admission date (in keeping with the Centers for Medicare & Medicaid Services definition of posthospitalization death) and postdischarge outcomes, conditional on surviving the index episode. Postdischarge outcomes included admission to a skilled nursing facility (SNF), defined as an SNF claim starting within 4 days of discharge from the index episode, and acute care visits (ED visits and/or observational stays and hospitalizations) within 90 days of discharge. Among index episodes, we quantified the occurrence of coding for cardiogenic shock and arrhythmias; utilization of ED, hospital, and intensive care services; total length of stay; and procedural interventions (including left heart catheterization, percutaneous coronary intervention, mechanical circulatory support, and invasive mechanical ventilation).

### Exposure

Our primary exposures were time period and beneficiary-level rurality. We defined time periods as prepandemic (January 1, 2018, to December 31, 2019), pandemic (January 1, 2020, to December 31, 2021), and postpandemic (January 1, 2022, to December 31, 2023). We defined beneficiary-level rurality using core-based statistical areas for metropolitan (hereinafter urban), micropolitan, and rural (not categorized as metropolitan or micropolitan) based on beneficiary county of residence Federal Information Processing Standards codes.^16^

All characteristics attributed to index AMI episodes are based on beneficiary-level values obtained at the index encounter date. Other relevant beneficiary-level characteristics were ascertained from the VRDC, including age, sex, race and ethnicity (using the Research Triangle Institute race code from the Master Beneficiary Summary File, which algorithmically augments self-reported race and ethnicity data [American Indian or Alaska Native, Asian or Pacific Islander, Black or African American, Hispanic, White, or other, including multiracial or a listed race not otherwise identified] from Social Security enrollment^17^), dual Medicaid enrollment, reason for Medicare eligibility, county-level quartile of social vulnerability index (2022 indices gathered from the Centers for Disease Control and Prevention and Agency for Toxic Substances and Disease Registry^18^), and preexisting conditions, based on Medicare’s Chronic Conditions Warehouse.^19^ Race and ethnicity were included because racial and ethnic disparities in cardiovascular care delivery and outcomes are well documented and may have worsened during the pandemic.

### Statistical Analysis

Patient characteristics, as well as all outcomes listed above, are reported as unadjusted rates or means, stratified by time period. Results are presented for all episodes, such that some patients may appear multiple times.

To illustrate the changes in STEMI and NSTEMI incidence during the 3 time periods, we calculated the quarterly rates of episodes of each diagnosis per million beneficiary days at risk and graphed them for beneficiaries residing in urban, micropolitan, and rural localities. To quantify the association of time period and time period by beneficiary-level rurality on select in-hospital outcomes, we fit logistic regression models to describe in-hospital death and death within 90 days of index admission, as well as admission to an SNF within 3 days of discharge conditional on surviving the index hospitalization. For posthospitalization outcomes, we fit quasi-Poisson regression models to describe the incidence of acute encounters per beneficiary quarter (ED visits and/or observational stays and inpatient stays) while allowing for extra-Poisson variability. In all models, the unit of analysis is each beneficiary episode, and the primary covariates were indicators for time period, rurality, and the interactions between time period and rurality. We fit all regression models using generalized estimating equations, clustering on hospitals, and included covariates describing beneficiary age, race and ethnicity, sex, rurality, and chronic conditions. Adjusted odds ratios (AORs) and adjusted incidence rate ratios (AIRRs) with 95% CIs presented by rurality in eTable 2 in Supplement 1 were derived using a linear combination of regression interaction terms. All *P* values for rurality by time terms are tests for the interaction term from our regression model assessing whether we have evidence to suggest that outcomes in our study period differ by level of rurality. More modeling details are given in the eAppendix in Supplement 1. To test the robustness of our findings, we performed separate post hoc sensitivity analyses excluding patients younger than 65 years and patients with preexisting ischemic heart disease.

All statistical analyses were performed using SAS Enterprise Guide, version 8.3 (SAS Institute Inc) within the VRDC environment, and all plots were produced using R, version 4.4.3 (R Program for Statistical Computing). To account for multiple comparisons and preserve a global type I error rate of 0.05, we used a Bonferroni correction to account for comparison of 30 interaction terms and as a result compare all *P* values to .002 (.05/30 comparisons) to ascertain statistical significance. All comparisons were 2 sided.

## Results

### Beneficiary-Level Characteristics Among Index AMI Episodes

Between January 2018 and December 2023, a total of 1 152 851 index AMI episodes occurred among 1 032 212 unique beneficiaries. NSTEMI accounted for 871 843 episodes (75.6%). More episodes occurred among male (663 553 [57.6%]) compared with female (498 318 [42.4%]) beneficiaries, with the most common age range being 65 to 80 years (654 6110 [56.8%]). In total, 9121 episodes (0.8%) occurred among American Indian or Alaska Native beneficiaries; 26 824 (2.3%), among Asian or Pacific Islander beneficiaries; 97 812 (8.5%), among Black or African American beneficiaries; 68 373 (5.9%), among Hispanic beneficiaries; 926 208 (80.3%), among White beneficiaries; 8555 (0.7%), among beneficiaries of other race or ethnicity; and 15 958 (1.4%), among beneficiaries whose race or ethnicity was unknown. Beneficiaries living in urban counties accounted for most episodes (858 071 [74.4%]), followed by micropolitan (160 065 [13.9%]) and rural (134 715 [11.7%]) counties. Prior ischemic heart disease was observed in 641 454 index episodes (55.6%), heart failure in 384 828 (33.4%), atrial fibrillation in 260 794 (22.6%), diabetes in 497 058 (43.1%), chronic kidney disease in 404 413 (35.1%), cancer in 168 643 (14.6%), and chronic obstructive pulmonary disease in 291 256 (25.3%). Comparing prepandemic with postpandemic episodes, beneficiaries in the later time periods were less often younger than 65 years, less often Black or African American, less often dually enrolled in Medicaid, and less often entitled to Medicare due to a disability (Table 1). The presence of key comorbidities was roughly stable across time periods.

**Table 1.** Patient-Level Characteristics Among Index AMI Episodes

Characteristic	Time period, No. (%) of episodes			
	Prepandemic (2018-2019) (n = 453 036)	Pandemic (2020-2021) (n = 366 249)	Postpandemic (2022-2023) (n = 333 566)	Overall (2018-2023) (N = 1 152 851)
No. of unique beneficiaries^a^	417 705	340 584	310 420	1 032 212
Age at time of index event, y				
<65	58 951 (13.0)	41 924 (11.4)	29 598 (8.9)	130 473 (11.3)
65-80	250 899 (55.4)	209 500 (57.2)	194 211 (58.2)	654 610 (56.8)
≥80	143 186 (31.6)	114 825 (31.4)	109 757 (32.9)	367 768 (31.9)
AMI subtype				
NSTEMI	343 466 (75.8)	274 751 (75.0)	253 626 (76.0)	871 843 (75.6)
STEMI	109 570 (24.2)	91 498 (25.0)	79 940 (24.0)	281 008 (24.4)
Sex				
Male	260 487 (57.5)	211 922 (57.9)	191 124 (57.3)	663 533 (57.6)
Female	192 549 (42.5)	154 327 (42.1)	142 442 (42.7)	489 318 (42.4)
Race or ethnicity^b^				
American Indian or Alaska Native	3631 (0.8)	2916 (0.8)	2574 (0.8)	9121 (0.8)
Asian or Pacific Islander	10 021 (2.2)	8357 (2.3)	8446 (2.5)	26 824 (2.3)
Black or African American	42 479 (9.4)	30 566 (8.3)	24 767 (7.4)	97 812 (8.5)
Hispanic	27 546 (6.1)	21 458 (5.9)	19 369 (5.8)	68 373 (5.9)
White	360 933 (79.7)	295 165 (80.6)	270 110 (81.0)	926 208 (80.3)
Other^c^	3235 (0.7)	2629 (0.7)	2691 (0.8)	8555 (0.7)
Unknown	5191 (1.1)	5158 (1.4)	5609 (1.7)	15 958 (1.4)
Dually enrolled in Medicaid	98 382 (21.7)	74 572 (20.4)	61 719 (18.5)	234 673 (20.4)
Reason for Medicare eligibility				
Age ≥65 y or survivor’s insurance	334 960 (73.9)	277 711 (75.8)	264 177 (79.2)	876 848 (76.1)
Disability	106 528 (23.5)	79 541 (21.7)	62 976 (18.9)	249 045 (21.6)
ESRD	6741 (1.5)	6055 (1.7)	4887 (1.5)	17 683 (1.5)
Disability and ESRD	4807 (1.1)	2942 (0.8)	1526 (0.5)	9275 (0.8)
Rurality (based on CBSA code)^d^				
Urban	338 174 (74.6)	271 696 (74.2)	248 201 (74.4)	858 071 (74.4)
Micropolitan	63 127 (13.9)	51 143 (14.0)	45 795 (13.7)	160 065 (13.9)
Rural	51 735 (11.4)	43 410 (11.9)	39 570 (11.9)	134 715 (11.7)
Social vulnerability index quartile^e^				
1 (Lowest vulnerability)	65 776 (14.5)	56 146 (15.3)	52 011 (15.6)	173 933 (15.1)
2	113 215 (25.0)	92 588 (25.3)	85 491 (25.6)	291 294 (25.3)
3	130 701 (28.9)	105 091 (28.7)	96 112 (28.8)	331 904 (28.8)
4 (Highest vulnerability)	143 344 (31.6)	112 424 (30.7)	99 952 (30.0)	355 720 (30.9)
Region				
Midwest	109 691 (24.2)	88 279 (24.1)	78 661 (23.6)	276 631 (24.0)
Northeast	75 689 (16.7)	60 031 (16.4)	55 291 (16.6)	191 011 (16.6)
South	188 864 (41.7)	151 325 (41.3)	134 833 (40.4)	475 022 (41.2)
West	78 792 (17.4)	66 614 (18.2)	64 781 (19.4)	210 187 (18.2)
Preexisting conditions				
Hypertension	349 517 (77.1)	280 208 (76.5)	252 110 (75.6)	881 835 (76.5)
Hyperlipidemia	329 572 (72.7)	271 710 (74.2)	251 205 (75.3)	852 487 (73.9)
Ischemic heart disease	253 466 (55.9)	203 425 (55.5)	184 563 (55.3)	641 454 (55.6)
Diabetes	199 566 (44.1)	157 904 (43.1)	139 588 (41.8)	497 058 (43.1)
Anemia	157 392 (34.7)	127 110 (34.7)	118 223 (35.4)	402 725 (34.9)
Chronic kidney disease	155 984 (34.4)	129 461 (35.3)	118 968 (35.7)	404 413 (35.1)
Heart failure	151 188 (33.4)	121 931 (33.3)	111 709 (33.5)	384 828 (33.4)
COPD	123 879 (27.3)	91 321 (24.9)	76 056 (22.8)	291 256 (25.3)
Atrial fibrillation	100 193 (22.1)	82 493 (22.5)	78 108 (23.4)	260 794 (22.6)
Depression	99 416 (21.9)	83 599 (22.8)	75 882 (22.7)	258 897 (22.5)
Cancer^f^	63 608 (14.0)	53 837 (14.7)	51 198 (15.3)	168 643 (14.6)
Stroke and TIA	59 156 (13.1)	40 051 (10.9)	40 604 (12.2)	139 811 (12.1)
Non-Alzheimer dementia	47 982 (10.6)	36 248 (9.9)	33 190 (10.0)	117 420 (10.2)
Alzheimer dementia	14 596 (3.2)	10 090 (2.8)	8573 (2.6)	33 259 (2.9)

Abbreviations: AMI, acute myocardial infarction; CBSA, core-based statistical area; COPD, chronic obstructive pulmonary disease; ESRD, end-stage renal disease; NSTEMI, non–ST-elevation myocardial infarction (STEMI); TIA, transient ischemic attack.

^a^ Some beneficiaries could have experienced an episode in 2 or 3 time periods and thus the total number of unique beneficiaries included in the cohort (from 2018-2023) is lower than the sum of the number of unique beneficiaries per period.

^b^ Identified using Research Triangle Institute Race Codes, which algorithmically augments self-reported race and ethnicity data from Social Security enrollment.

^c^ Includes individuals who identified as multiracial or as a listed race not otherwise identified.

^d^ Delineates counties as metropolitan (populations >50 000), micropolitan (populations 10 000-50,000), or rural (neither metropolitan or micropolitan).

^e^ Quantifies degrees of social vulnerability based on a community’s socioeconomic status, household characteristics, race and ethnicity, housing, and transportation. Data were gathered from 2022.

^f^ Includes breast, colorectal, endometrial, lung, prostate, and urothelial diagnoses.

### Index AMI Episode Characteristics

**Figure 1.**
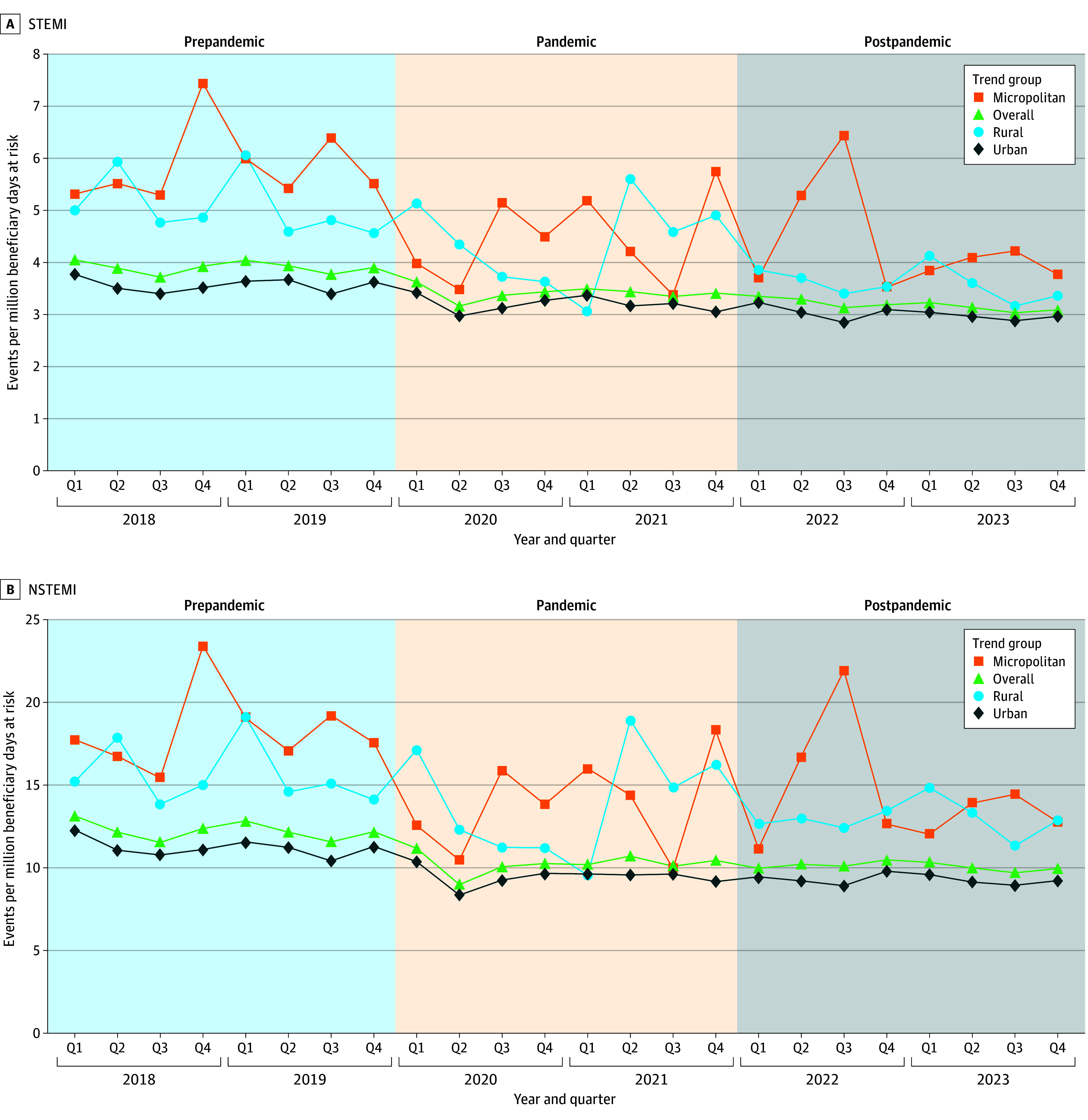
Frequency Polygon Graphs Showing Unadjusted Incidence of ST-Segment Elevation Myocardial Infarction (STEMI) and Non-STEMI (NSTEMI) Among Fee-for-Service Medicare Beneficiaries Data are stratified by beneficiary rurality. Across the study period, the unadjusted incidence of STEMI decreased from 4.05 (quarter 1 [Q1] of 2018) to 3.09 episodes per million beneficiary days at risk (quarter 4 [Q4] of 2023). The incidence of NSTEMI decreased from 13.13 (Q1 of 2018) to 9.95 episodes per million beneficiary days at risk (Q4 of 2023).

The incidence of index AMI episodes decreased from 2018 to 2023 (Figure 1). The unadjusted quarterly incidence of AMI decreased from 17.2 (quarter 1 of 2018) to 13.0 (to quarter 4 of 2023) episodes per million beneficiary days at risk. The incidence of STEMI decreased from 4.05 (quarter 1 of 2018) to 3.09 (quarter 4 of 2023) episodes per million beneficiary days at risk; NSTEMI decreased from 13.13 (quarter 1 of 2018) to 9.95 (quarter 4 of 2023) episodes per million beneficiary days at risk.

Episode characteristics by time period are detailed in Table 2. Coding for cardiogenic shock increased from 31 356 of 453 036 (6.9%) to 25 487 of 333 566 episodes (7.6%), and arrythmias increased from 196 585 of 453 036 (3.4%) to 158 929 of 333 566 episodes (47.7%). The overall mean (SD) length of stay was 6.1 (7.3) days with little change across periods. Utilization of the intensive care unit decreased across periods, from 317 851 of 453 036 episodes (70.2%) before the pandemic to 219 444 of 333 566 (65.8%) after the pandemic. Left heart catheterization and percutaneous coronary intervention occurred in 684 803 (59.4%) and 420 371 (36.5%) of 1152 851 episodes, respectively, and invasive mechanical ventilation was used in 77 329 (6.7%) of 1 152 851 episodes across the study period, without major temporal changes. Other procedures were coded in a smaller proportion of episodes, including intra-aortic balloon pump (1786 [0.2%]), Impella catheter insertion (20 955 [1.8%]), extracorporeal membrane oxygenation (2226 [0.2%]), and left ventricular assist device implantation (861 [0.1%]).

**Table 2.** Index AMI Episode Characteristics

Characteristic	Time period, No. (%) of episodes			
	Prepandemic (2018-2019) (n = 453 036)	Pandemic (2020-2021) (n = 366 249)	Postpandemic (2022-2023) (n = 333 566)	Overall (2018-2023) (N = 1 152 851)
AMI subtype				
STEMI	109 570 (24.2)	91 498 (25.0)	79 940 (24.0)	281 008 (24.4)
NSTEMI	343 466 (75.8)	274 751 (75.0)	253 626 (76.0)	871 843 (75.6)
Complications				
Cardiogenic shock	31 356 (6.9)	26 471 (7.2)	25 487 (7.6)	83 314 (7.2)
Arrhythmia	196 585 (43.4)	168 455 (46.0)	158 929 (47.7)	523 969 (45.4)
Index episode hospital utilization				
ED utilization	426 422 (94.1)	347 574 (94.9)	317 494 (95.2)	1 091 490 (94.7)
Inpatient hospitalization	424 502 (93.7)	339 180 (92.6)	307 608 (92.2)	1 071 290 (92.9)
ICU utilization	317 851 (70.2)	245 098 (66.9)	219 444 (65.8)	782 393 (67.9)
Total length of stay, mean (SD), d	6.2 (7.3)	6.0 (7.3)	6.1 (7.5)	6.1 (7.3)
Procedural interventions				
Left heart catheterization	268 058 (59.2)	217 691 (59.4)	199 054 (59.7)	684 803 (59.4)
Percutaneous coronary intervention	163 561 (36.1)	135 343 (37.0)	121 467 (36.4)	420 371 (36.5)
Intra-aortic balloon pump insertion	773 (0.2)	561 (0.2)	452 (0.1)	1786 (0.2)
Impella catheter insertion	7297 (1.6)	6661 (1.8)	6997 (2.1)	20 955 (1.8)
ECMO^a^	596 (0.1)	775 (0.2)	855 (0.3)	2226 (0.2)
LVAD implantation	266 (0.1)	278 (0.1)	317 (0.1)	861 (0.1)
IMV ≤96 h	22 654 (5.0)	18 518 (5.1)	16 477 (4.9)	57 649 (5.0)
IMV >96 h	7887 (1.7)	6459 (1.8)	5334 (1.6)	19 680 (1.7)

Abbreviations: AMI, acute myocardial infarction; ECMO, extracorporeal membrane oxygenation; ED, emergency department; ICU, intensive care unit; IMV, invasive mechanical ventilation; LVAD, left ventricular assist device; NSTEMI, non–ST-elevation myocardial infarction (STEMI).

^a^ Includes central and peripheral venovenous and venoarterial cannulation.

### In-Hospital Death

**Figure 2.**
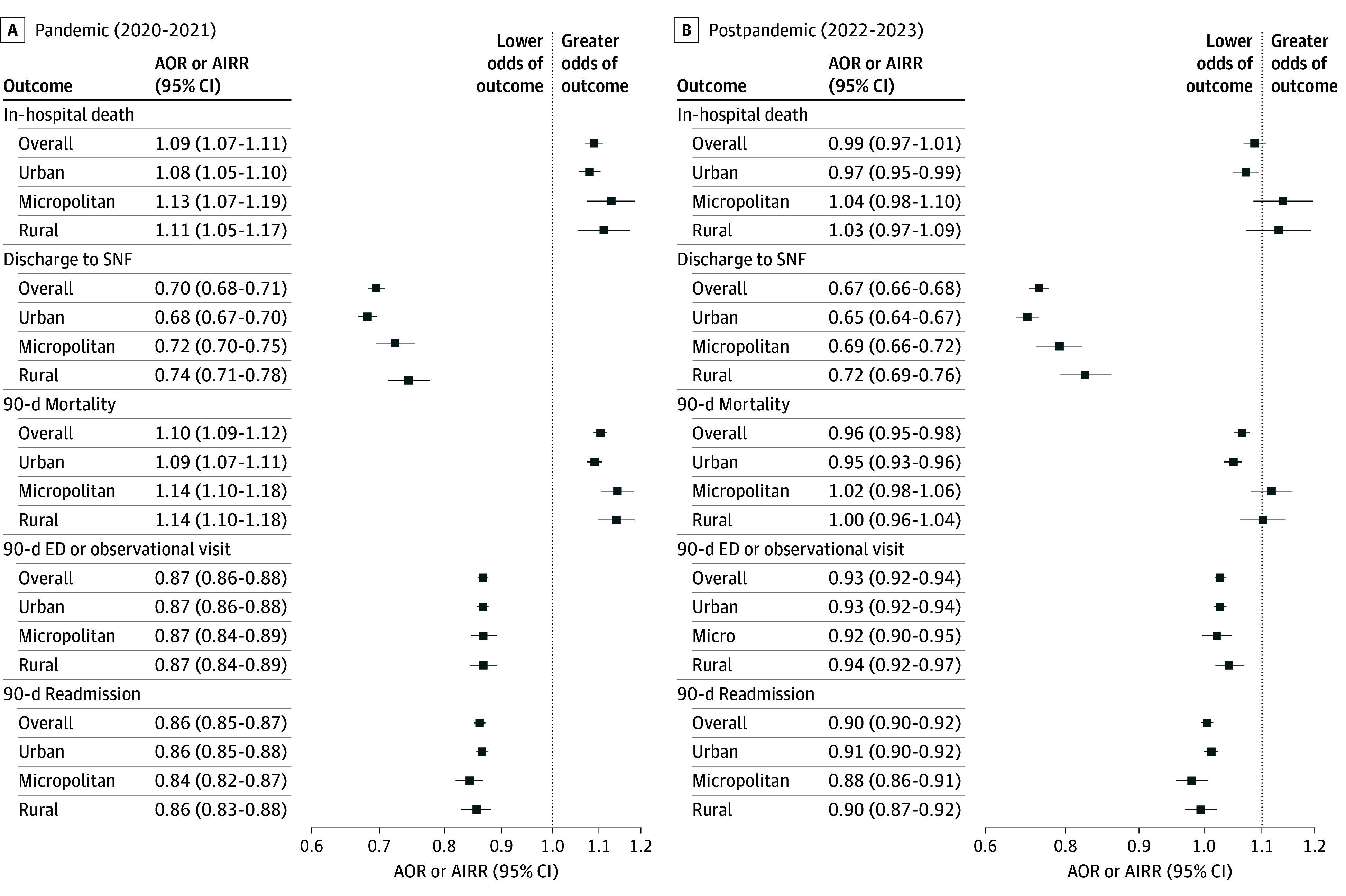
Subgroup Analyses Showing Acute Myocardial Infarction Episode Outcomes During and After the Pandemic Adjusted odds ratios (AORs) for in-hospital death and discharge to skilled nursing facility (SNF) and adjusted incidence rate ratios (AIRRs) for 90-day mortality, 90-day emergency department (ED) or observational visits, and 90-day readmission were calculated using prepandemic (2018-2019) outcomes as a reference.

In-hospital death occurred during 31 037 index AMI episodes (6.9%) before the pandemic, 27 266 (7.4%) during the pandemic, and 23 557 (7.1%) after the pandemic (Table 3). With the prepandemic period as a reference, there were greater odds of in-hospital death during the pandemic (AOR, 1.09; 95% CI, 1.07-1.11), with similar increases across beneficiaries by rurality (Figure 2); after the pandemic, the odds of in-hospital death overall were not different from those before the pandemic (AOR, 0.99; 95% CI, 0.97-1.01), although they were lower among urban beneficiaries (AOR, 0.97; 95% CI, 0.95-0.99).

**Table 3.** AMI Outcomes

Outcome	Time period, No. (%) of episodes			
	Prepandemic (2018-2019) (n = 453 036)	Pandemic (2020-2021) (n = 366 249)	Postpandemic (2022-2023) (n = 333 566)	Overall (2018-2023) (n = 1 152 851)
Index AMI episode outcome				
Death	31 037 (6.9)	27 266 (7.4)	23 557 (7.1)	81 860 (7.1)
Discharge to SNF	51 827 (11.4)	30 232 (8.3)	27 838 (8.4)	109 897 (9.5)
90-d Outcome^a^				
ED visit and/or observational stay, mean (SD)	0.8 (1.4)	0.7 (1.3)	0.7 (1.2)	0.7 (1.3)
Inpatient hospitalization, mean (SD)	0.4 (0.8)	0.4 (0.7)	0.4 (0.8)	0.4 (0.8)
Total length of stay, mean (SD)^b^	3.6 (9.2)	3.1 (44.7)	3.2 (8.8)	3.3 (26.3)
Death	70 070 (15.5)	60 698 (16.6)	51 298 (15.4)	182 066 (15.8)

Abbreviations: AMI, acute myocardial infarction; ED, emergency department; SNF, skilled nursing facility.

^a^ Surveillance for postdischarge death began on the date of admission, in keeping with the Centers for Medicare & Medicaid Services definition of posthospitalization death. Surveillance for remaining postdischarge outcomes began on the date of discharge from the index AMI episode.

^b^ Measured among beneficiaries who survived an index AMI episode.

### Discharge to SNF

Discharge to an SNF decreased across the study period, from 51 827 (11.4%) before the pandemic to 27 838 (8.4%) after the pandemic (Table 3). There were lower odds of discharge to an SNF overall during (AOR, 0.70; 95% CI, 0.68-0.71) and after (AOR, 0.67; 95% CI, 0.66-0.68) the pandemic. Urban beneficiaries were significantly less likely than rural beneficiaries to be discharged to an SNF during the pandemic (AORs, 0.68 [95% CI, 0.67-0.70] vs 0.74 [95% CI, 0.71-0.78], respectively; *P* < .001 for the interaction) and after the pandemic (AORs, 0.65 [95% CI, 0.64-0.67] vs 0.72 [95% CI, 0.69-0.76], respectively; *P* < .001 for the interaction) (Figure 2 and eTable 2 in Supplement 1).

### Ninety-Day Mortality

Death within 90 days of the index AMI episode occurred among 70 070 episodes (15.5%) before the pandemic, 60 698 (16.6%) during the pandemic, and 51 298 (15.4%) after the pandemic (Table 3). There were higher odds of 90-day mortality overall during the pandemic (AOR, 1.10; 95% CI, 1.09-1.12) with similar increases across beneficiaries by rurality (Figure 2). After the pandemic, there were lower odds of 90-day mortality overall (AOR, 0.96; 95% CI, 0.95-0.98) but only among urban beneficiaries (AOR, 0.95; 95% CI, 0.93-0.96) and not micropolitan (AOR, 1.02; 95% CI, 0.98-1.06) or rural (AOR, 1.00; 95% CI, 0.96-1.04) beneficiaries.

### Ninety-Day Acute Care

Among beneficiaries who survived the index AMI episode, the mean (SD) number of ED visits and/or observational stays within 90 days of discharge trended from 0.8 (1.4) before the pandemic to 0.7 (1.2) during and after the pandemic. There were lower rates of ED visits and/or observational stays overall during the pandemic (AIRR, 0.87; 95% CI, 0.86-0.88) and after the pandemic (AIRR, 0.93; 95% CI, 0.92-0.94) with similar rates across beneficiaries by rurality.

The mean (SD) number of readmissions within 90 days of discharge was 0.4 (0.8) overall and was similar across time periods. With the prepandemic period as a reference, there were lower rates of readmission overall both during the pandemic (AIRR, 0.86; 95% CI, 0.85-0.87) and after the pandemic (AIRR, 0.90; 95% CI, 0.90-0.92) with similar rates across beneficiaries by rurality.

### Post Hoc Sensitivity Analyses

After excluding patients with a history of ischemic heart disease, the odds of in-hospital death remained higher during the pandemic (AOR, 1.10; 95% CI, 1.07-1.13) and returned to baseline after the pandemic (AOR, 1.02; 95% CI, 0.99-1.05) with no difference by rurality. Similarly, after excluding patients younger than 65 years, the odds of in-hospital death remained higher during the pandemic (AOR, 1.07; 95% CI, 1.05-1.09) and returned to baseline after the pandemic (AOR, 0.98; 95% CI, 0.96-1.00), with no difference by rurality (eTable 3 in Supplement 1).

## Discussion

In this nationwide analysis of more than 1 million Medicare FFS beneficiaries, the incidence of AMI declined from 2018 to 2023. Beneficiaries across geographies experienced higher in-hospital and 90-day mortality during the pandemic. After the pandemic, mortality improved among urban beneficiaries but only returned to baseline for micropolitan and rural beneficiaries. Discharges to SNFs declined markedly across the study period, with the largest decreases among urban beneficiaries. Return ED visits, observational stays, and readmissions also declined throughout the pandemic and postpandemic periods.

Our first important finding was the sustained decline in AMI incidence beginning early in the pandemic. Declines in AMI were previously reported during the pandemic,^4,6,20^ and ED visits sharply declined among Medicare beneficiaries beginning in March 2020, even for critical conditions.^21^ Fear of infectious exposure and resulting delays in presentation (or lack of presentation entirely) may have contributed to early reductions in ED visits; however, the persistent decline in incidence that we observed suggests additional explanations. COVID-19 caused substantial excess mortality among Medicare beneficiaries and may have altered the risk profile among remaining beneficiaries, leading to a lower incidence after the pandemic.^22,23^ An alternative explanation is shifting risk profiles between FFS and Medicare Advantage (MA) beneficiaries. As of 2022, half of all Medicare beneficiaries were enrolled in MA,^24^ and while MA beneficiaries were historically considered healthier and at lower risk than FFS beneficiaries, recent data suggest similar cardiovascular risk and outcomes between the 2 groups in part driven by this ongoing shift in enrollment.^25^

Our second finding was greater in-hospital and 90-day mortality during the pandemic. Other studies have observed greater mortality from ischemic heart disease during the COVID-19 pandemic^6,26,27^ as well as greater mortality from related conditions, including diabetes and stroke.^28,29^ The increase in cardiovascular mortality during the pandemic may have been due to disruptions in diagnostic and therapeutic procedures and delays in presentation owing to fear of infection and stay-at-home precautions.^30,31^ Our study is the first, to our knowledge, to find that AMI-related mortality returned to or decreased below the prepandemic baseline after the COVID-19 pandemic, supporting the hypothesis that pandemic-related care disruptions were a significant driver of higher cardiovascular mortality during the pandemic.

We observed lower rates of discharge to SNF during the pandemic that continued after the pandemic, particularly among urban beneficiaries. The pandemic was devastating to SNFs, both in terms of residents and staff; in many ways, SNFs were the epicenter of the early COVID-19 phenomenon. Early in COVID-19, there were high levels of fear about being discharged to an SNF, and many families chose to take their loved ones home rather than expose them to COVID-19 at a care facility. Later in the pandemic, there was a nationwide decline in SNF capacity, as SNFs struggled to retain staff and funding. As recently as 2023, SNFs were the last remaining care sector that had not met or exceeded prepandemic capacity.^32^ Whether these decreases in SNF utilization are associated with any detrimental changes in long-term functional capacity is an important area for future work.

We observed several differences between urban and rural beneficiaries; in particular, urban beneficiaries experienced a significant reduction in mortality after the pandemic, unlike micropolitan and rural beneficiaries.^25^ Disparate access to chronic care in rural areas owing to physician shortages, travel distance, and other social determinants of health have been well characterized and may have contributed to the differential changes in mortality.^33^ Previous work observed rising ED utilization among rural relative to urban US residents, owing to less access to outpatient services in rural areas, further supporting this as an important social determinant of health.^34^ Addressing disparate mortality in rural areas will require attention to a wide range of social and structural drivers of health outcomes.

### Limitations

Our study has several important limitations. First, while the nationwide scope of our dataset enhances the generalizability of our findings, FFS beneficiaries differ systematically from MA beneficiaries and privately insured individuals.^24^ Further, beneficiaries younger than 65 years are a unique and higher-risk population, owing to their qualifying conditions; therefore, our study findings do not fully evaluate the trends in AMI among younger patients more broadly, the incidence of which increased between 2008 and 2019.^35^ Second, claims data rely on the accuracy of coding and documentation, and because our analysis relied on billing claims, we were unable to capture AMIs that led to death without contact with medical care, a phenomenon that might have increased during the COVID-19 pandemic. Third, while our chosen analysis method allowed us to characterize and adjust for baseline risk as a function of beneficiary characteristics, it did not give us the flexibility to attribute changes in outcomes to the dynamic temporal processes that the COVID-19 pandemic followed. These results should be interpreted as broadly descriptive averages across large time periods across the COVID-19 pandemic rather than changes attributable to specific COVID-19 waves. Additionally, logistic regression during fixed follow-up time windows is how the Centers for Medicare & Medicaid Services approaches risk-adjusted mortality measures, which allowed us to include individual-level risk characteristics and incorporate within-hospital outcome correlation in our modeling strategy. We acknowledge that this methodology may fail to characterize nuances in differential survival time by group outside the 30- or 90-day follow-up periods chosen, which could be better explored using time-to-event models.

## Conclusions

In this retrospective cohort study of all FFS Medicare beneficiaries, the incidence of AMI declined from 2018 to 2023. In-hospital and 90-day mortality increased during the pandemic and improved below baseline after the pandemic for urban but not micropolitan or rural beneficiaries. Beneficiaries saw reductions in acute care, including readmission, and SNF utilization during and after the pandemic. Further work is needed to better understand the changing cardiovascular risk profiles among Medicare beneficiaries and to seek innovative solutions to the disparate outcomes among nonurban beneficiaries.

## Notes

Supplement 1. **eTable 1. ***ICD-10* Diagnostic and Procedure Codes **eFigure. **Rules Defining Care Encounters Among Fee-for-Service Medicare Beneficiaries **eTable 2. **Primary and Secondary Outcome OR and IRR Overall and by Rurality **eTable 3. **Post Hoc Sensitivity Analyses **eAppendix. **Technical Appendix

Supplement 2.
Data Sharing Statement
