# Supplementary material for: Acute Myocardial Infarction in Medicare Beneficiaries During and After the COVID-19 Pandemic
Source: JAMA Netw Open. 2026 Apr 1;9(4):e264122. doi: 10.1001/jamanetworkopen.2026.4122 (PMC13044675; doi:10.1001/jamanetworkopen.2026.4122)
Supplement: Supplement 1. — eTable 1. ICD-10 Diagnostic and Procedure Codes eFigure. Rules Defining Care Encounters Among Fee-for-Service Medicare Beneficiaries eTable 2. Primary and Secondary Outcome OR and IRR Overall and by Rurality eTable 3. Post Hoc Sensitivity Analyses eAppendix. Technical Appendix [file jamanetwopen-e264122-s001.pdf]

Supplementary Online Content

Graves J, Waken RJ, Wang F, et al. Acute myocardial infarction in Medicare beneficiaries during and after the COVID-19 pandemic. *JAMA Netw Open.* 2026;9(4):e264122. doi:10.1001/jamanetworkopen.2026.4122

eTable 1. *ICD-10* Diagnostic and Procedure Codes

eFigure. Rules Defining Care Encounters Among Fee-for-Service Medicare Beneficiaries

eTable 2. Primary and Secondary Outcome OR and IRR Overall and by Rurality

eTable 3. Post Hoc Sensitivity Analyses

eAppendix. Technical Appendix

This supplementary material has been provided by the authors to give readers additional information about their work.

© 2026 Graves J et al. *JAMA Network Open*.

eTable 1. *ICD-10* Diagnostic and Procedure Codes

**Diagnosis codes**	
ST-elevated myocardial infarction	I210, I2101, I2102, I2109, I211, I2111, I2119, I212, I2121, I2129, I213
Non-ST-elevated myocardial infarction	I214
Cardiogenic shock	R570
Arrythmia	I490, I4901, I4902, I491, I492, I493, I494, I4940, I4949, I495, I498, I499, I440, I441, I442, I443, I4430, I4439, I444, I445, I446, I4460, I4469, I447, I450, I451, I4510, I4519, I452, I453, I454, I455, I456, I458, I4581, I4589, I459, I470, I471, I4710, I4711, I4719, I472, I4720, I4721, I4729, I479, I480, I481, I4811, I4819, I482, I4820, I4821, I483, I484, I489, I4891, I4892
**Procedure Codes**	
Left heart catheterization	4A023N6, 4A023N7, 4A023N8
Percutaneous coronary intervention	0270346, 027034Z, 0270356,027035Z,0270366, 027036Z, 0270376, 027037Z, 02703D6, 02703DZ, 02703E6, 02703EZ, 02703F6, 02703FZ,02703G6, 02703GZ, 02703T6,02703TZ, 02703ZZ, 02713ZZ, 02723ZZ, 02733ZZ
Intra-aortic balloon pump	5A02110,5A02216
Impella	5A0221D
Extra-corporeal membrane oxygenation	5A1522F, 5A1522G, 5A1522H ,5A15A23, 5A15A2F
Left ventricular assist device	02HA0QZ, 02HA0RJ, 02HA0RS,02HA0RZ, 02HA0YZ
Invasive mechanical ventilation ≤96 hours	5A1935Z, 5A1945Z
Invasive mechanical ventilation >96 hours	5A1955Z

ICD-10, *International Classifications of Diseases* 10th revision

eFigure. Rules Defining Care Encounters Among Fee-for-Service Medicare Beneficiaries

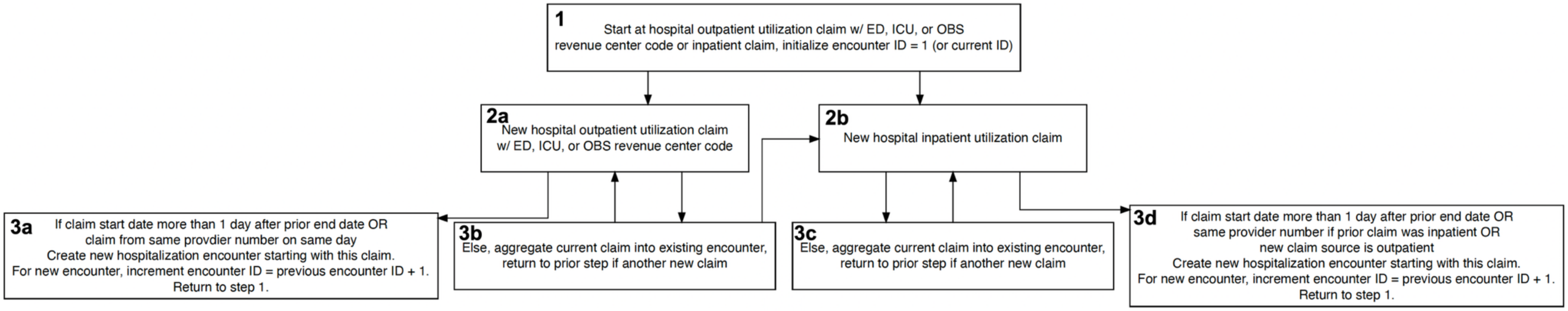

ED, emergency department; ICU, intensive care unit; ID, identification; OBS, observational stay

Created in https://BioRender.com.

© 2026 Graves J et al. *JAMA Network Open*.

eTable 2. Primary and Secondary Outcome OR and IRR Overall and by Rurality

	Time	Level	AOR/AIRR (95% CI)	Test	*P* value[a]
Death during index AMI hospitalization	2020 - 2021 vs 2018 - 2019	Overall	1.09 (1.07 - 1.11)	Overall Change	<0.0001
		Urban	1.08 (1.06 - 1.10)	Reference	---
		Micropolitan	1.13 (1.07 - 1.19)	Time x Micro Interaction	0.1155
		Rural	1.11 (1.05 - 1.17)	Time x Rural Interaction	0.3161
	2022 - 2023 vs 2018 - 2019	Overall	0.99 (0.98 - 1.04)	Overall Change	0.1871
		Urban	0.97 (0.95 - 0.99)	Reference	---
		Micropolitan	1.04 (0.99 - 1.10)	Time x Micro Interaction	0.0198
		Rural	1.03 (0.97 - 1.09)	Time x Rural Interaction	0.0614
Discharge to SNF	2020 - 2021 vs 2018 - 2019	Overall	0.70 (0.68 - 0.71)	Overall Change	<0.0001
		Urban	0.68 (0.67 - 0.70)	Reference	---
		Micropolitan	0.72 (0.70 - 0.75)	Time x Micro Interaction	0.0099
		Rural	0.74 (0.71 - 0.78)	Time x Rural Interaction	0.0003
	2022 - 2023 vs 2018 - 2019	Overall	0.67 (0.66 - 0.68)	Overall Change	<0.0001
		Urban	0.65 (0.64 - 0.67)	Reference	---
		Micropolitan	0.69 (0.66 - 0.72)	Time x Micro Interaction	0.0120
		Rural	0.73 (0.69 - 0.76)	Time x Rural Interaction	<0.0001
Death within 90 days of index AMI admission	2020 - 2021 vs 2018 - 2019	Overall	1.10 (1.09 -1.12)	Overall Change	<0.0001
		Urban	1.09 (1.07 - 1.11)	Reference	---
		Micropolitan	1.14 (1.11 - 1.18)	Time x Micro Interaction	0.0120
		Rural	1.14 (1.10 - 1.18)	Time x Rural Interaction	0.0269
	2022 - 2023 vs 2018 - 2019	Overall	0.96 (0.95 - 0.98)	Overall Change	<0.0001
		Urban	0.95 (0.93 - 0.96)	Reference	---
		Micropolitan	1.02 (0.98 - 1.06)	Time x Micro Interaction	0.0007
		Rural	1.00 (0.96 - 1.04)	Time x Rural Interaction	0.0151
ED visits and/or observational stay within 90 days of discharge	2020 - 2021 vs 2018 - 2019	Overall	0.87 (0.86 - 0.88)	Overall Change	<0.0001
		Urban	0.87 (0.86 - 0.88)	Reference	---
		Micropolitan	0.87 (0.85 - 0.89)	Time x Micro Interaction	0.9004
		Rural	0.87 (0.84 - 0.89)	Time x Rural Interaction	0.9328

© 2026 Graves J et al. *JAMA Network Open*.

	2022 - 2023 vs 2018 - 2019	Overall	0.93 (0.92 - 0.94)	Overall Change	<0.0001
		Urban	0.93 (0.92 - 0.94)	Reference	---
		Micropolitan	0.92 (0.90 - 0.95)	Time x Micro Interaction	0.7121
		Rural	0.94 (0.92 - 0.97)	Time x Rural Interaction	0.2191
Hospitalization within 90 days of discharge	2020 - 2021 vs 2018 - 2019	Overall	0.86 (0.85 - 0.87)	Overall Change	<0.0001
		Urban	0.87 (0.85 - 0.88)	Reference	---
		Micropolitan	0.84 (0.82 - 0.87)	Time x Micro Interaction	0.1117
		Rural	0.86 (0.83 - 0.88)	Time x Rural Interaction	0.4816
	2022 - 2023 vs 2018 - 2019	Overall	0.91 (0.90 - 0.92)	Overall Change	<0.0001
		Urban	0.91 (0.90 - 0.92)	Reference	---
		Micropolitan	0.88 (0.86 - 0.91)	Time x Micro Interaction	0.0294
		Rural	0.90 (0.87 - 0.92)	Time x Rural Interaction	0.2530

AMI, acute myocardial infarction; CI, confidence interval; FFS, fee-for-service; AIRR, adjusted incidence rate ratio; Micro, micropolitan; AOR, adjusted odds ratio; SNF, skilled nursing facility

[a] $P < 0.0017$ is considered significant in order to account for multiple comparisons and preserve a global type I error rate of 0.05.

© 2026 Graves J et al. *JAMA Network Open*.

eTable 3. Post Hoc Sensitivity Analyses

Outcome	Time	Level	AOR (95% CI)
**Excluding patients with a history of ischemic heart disease**			
Death during index AMI hospitalization	2020-2021 vs 2018-2019	Overall	1.10 (1.07 - 1.13)
		Urban	1.10 (1.06 - 1.14)
		Micropolitan	1.09 (1.01 - 1.17)
		Rural	1.16 (1.07 - 1.25)
	2022-2023 vs 2018-2019	Overall	1.02 (0.99 - 1.05)
		Urban	1.00 (0.97 - 1.04)
		Micropolitan	1.04 (0.96 - 1.12)
		Rural	1.06 (0.97 - 1.16)
**Excluding patients <65 years of age**			
Death during index AMI hospitalization	2020-2021 vs 2018-2019	Overall	1.07 (1.05 - 1.09)
		Urban	1.07 (1.05 - 1.1)
		Micropolitan	1.12 (1.06 - 1.18)
		Rural	1.11 (1.05 - 1.17)
	2022-2023 vs 2018-2019	Overall	0.98 (0.96 – 1.00)
		Urban	0.97 (0.95 - 0.99)
		Micropolitan	1.02 (0.97 - 1.08)
		Rural	1.02 (0.96 - 1.08)
AMI, acute myocardial infarction; AOR, adjusted odds ratio			

© 2026 Graves J et al. *JAMA Network Open*.

eAppendix. Technical Appendix

Model specification

Generally, we specify one of two different sets of regression predictors for each of our models; in models where we investigate an overall effect, we specify

$$E[y_i] = f^{-1}(\beta_0 + \boldsymbol{x}_{i,P}\boldsymbol{\beta}_P + \boldsymbol{x}_{i,R}\boldsymbol{\beta}_R + \boldsymbol{x}_{i,S}\boldsymbol{\beta}_S + \boldsymbol{x}_{i,D}\boldsymbol{\beta}_D + \boldsymbol{x}_{i,C}\boldsymbol{\beta}_C),$$

where $\boldsymbol{x}_{i,P}$ is the vector describing the time-period predictor categorical variables and $\boldsymbol{\beta}_P$ are the coefficients of inferential interest, $\boldsymbol{x}_{i,R}$ is the vector of predictors describing levels of rurality (micropolitan vs urban, rural vs urban), $\boldsymbol{x}_{i,D}$ is the vector of demographic predictors for age (<65, 65-74, 75-84, 85+), race (White, Black, Hispanic, Other), and sex (Male, Female) groups, $\boldsymbol{x}_{i,C}$ is the vector of chronic condition flags that align with the chronic conditions referenced in the methods section, $x_{i,S}$ is vector of severity and complication flags, including any ST-elevated myocardial infarction (STEMI) diagnosis (vs non-ST-elevated myocardial infarction only), cardiogenic shock diagnosis, and arrythmia diagnosis, and $\boldsymbol{\beta}_R$, $\boldsymbol{\beta}_D$, $\boldsymbol{\beta}_S$, and $\boldsymbol{\beta}_C$ are the regression coefficients estimated simultaneously with our coefficients of interest to achieve adjusted results.

In models where we investigate differential effects by levels of rurality, we specify the model

$$E[y_i] = f^{-1}(\beta_0 + \boldsymbol{x}_{i,P}\boldsymbol{\beta}_P + \boldsymbol{x}_{i,R}\boldsymbol{\beta}_R + \boldsymbol{x}_{i,S}\boldsymbol{\beta}_S + \boldsymbol{x}_{i,P\times R}\boldsymbol{\beta}_{P\times R} + \boldsymbol{x}_{i,D}\boldsymbol{\beta}_D + \boldsymbol{x}_{i,C}\boldsymbol{\beta}_C),$$

where we simply add an interaction term between rurality in time-period to our set of predictors with $\boldsymbol{x}_{i,P\times R}$, and our inferences of interest involve $\boldsymbol{\beta}_{P\times R}$.

In models that describe counts of utilization based measures 90 days post-index hospitalization discharge, we add offset terms to the above models to account for beneficiaries leaving the sample via death or disenrollment from fee-for-service Medicare.

Inferences about pre-post changes across levels of rurality

In order to describe interpretable changes in utilization and mortality across time periods within levels of rurality, we use linear combinations of coefficients estimated using the aforementioned models; this ensures a consistent set of inferences and interpretations for each of our claims. We achieve this in the SAS GENMOD procedure using the estimate statement. In all cases pre-post changes in utilization or mortality within levels of rurality, we present confidence intervals for that change within each level of rurality, and *P* values describing the test of the interaction term, which immediately helps readers ascertain whether we have evidence to claim that the pre-post change was different in micropolitan or rural areas when compared back to urban areas.

© 2026 Graves J et al. *JAMA Network Open*.

Fitting specifics

Fitting models to large datasets containing millions of rows is difficult, even for software like SAS that can mitigate some computational complexity with disk and memory swaps. To facilitate these model fits, we work with sufficient statistics instead of each individual hospitalization claim where we sum up mortality, the number of index hospitalizations, the number of post-hospitalization days at risk, and all outcome measures of interest at the intersection of all covariates and hospitals in the models listed out above; we then use the GENMOD procedure to fit models using the generalized estimating equations, setting the within cluster correlation parameter to update every four iterations; this allows the model to converge, and borders on conservatism (i.e., inflated standard errors) as the within cluster correlation is initialized at the Huber-White sandwich estimator value. All models are checked for convergence before using outputs for inference.

© 2026 Graves J et al. *JAMA Network Open*.
