# Supplementary material for: Acute Myocardial Infarction in Medicare Beneficiaries During and After the COVID-19 Pandemic
Source: JAMA Netw Open. 2026 Apr 1;9(4):e264122. doi: 10.1001/jamanetworkopen.2026.4122 (PMC13044675; doi:10.1001/jamanetworkopen.2026.4122)
Supplement: Supplement 2. — Data Sharing Statement [file jamanetwopen-e264122-s002.pdf]

Data Sharing Statement

Graves. Acute Myocardial Infarction in Medicare Beneficiaries During and After the COVID-19 Pandemic. *JAMA Netw Open*. Published April 01, 2026.
doi:10.1001/jamanetworkopen.2026.4122

Data
Data available: No

Additional Information
Explanation for why data not available: Data are accessed through a secure online federal portal and are not possible to share. Interested researchers can apply to access the data directly through CMS.
